# Twelve Hundred Miles under Difficulties

**Published:** 1889-04-20

**Authors:** Daniel Chamier


### Twelve Hundred Miles under Difficulties.

By Daniel Chamier.

Most men and women are seized by an impulse at one time or another; indeed, I think it has been said that we are the creatures of impulse—poor, frail mortals that we are. At any rate, not so long ago, I developed a violent ambition to visit the South Sea Islands, so I looked about the harbour of Auckland, New Zealand, for a vessel that would take me thither. The only craft in which any hope could be placed was a small schooner of 62 tons, rejoicing in the name of "Toafa Haamia," and even she could hardly be looked to with much certainty. She was still on the stocks, being in course of completion, and had been ordered especially for the Tongan Government by Mr. Baker, the missionary premier. The builders had contracted with a qualified master to have her delivered for a specified sum, and it suited the skipper well enough to carry a passenger or two as well. But the Customs House regulations forbade the latter course, and the only alternative left me was to ship as steward. This I consented to do, and having paid my passage money, duly signed the articles. The boat was launched, ballasted, and fitted out, and the day of departure arrived. I had omitted to make enquiries as to the crew, never supposing that such precautions would be necessary; so I stepped innocently aboard the schooner as she lay gently heaving to and fro in the lovely harbour of Auckland. As it was a voyage of surprises and novel experiences, I soon settled into the philosophic method of taking everything as it came; but the first astonishment amounted to something approaching a shock, for I found that the "crew" consisted of the following brilliant array of humanity: (1) The skipper's boy; (2) a young lad of sixteen; (3) two broken-down music hall singers of the most miserable type; (4) the wife of one of them; (5) a six months' old baby. These, with the skipper, were to navigate the vessel over a difficult course of 1,200 miles, through boisterous trade winds (and perhaps hurricanes), in and out of coral reefs, shoals, and islands. There was no time to deliberate. I was determined to go, and that settled the matter. We heaved up the anchor—as much as we could do—hoisted the sails one by one, by dint of much tugging and hauling, and departed.

Auckland gradually dropped astern, and the lovely green hills grew greyer and fainter, and then we were surrounded by sea alone. The cause of this rapid departure was a spanking gale of wind, which sprung up aft and drove us headlong through the sea, the lee-scuppers dipping under water, and every rope creaking and every sail taut. It was then discovered for the first time that we were (1) under-ballasted, (2) over-masted, (3) barely provisioned, (4) provided with a miserably inaccurate compass, (5) with a chronometer that kept very little better time than the 10s. 6d. Waterbury with which I had carefully provided myself before starting. Though the breeze stiffened, we dared not haul down the mainsail, because, as the skipper remarked, "Lor'! we'd never get it up again!" So we clung to the deck—at an angle of 45deg.—kept our course as best we could, sent the boys to bed (after the skipper had somewhat eased his feelings by thrashing them), and divided the watches as best we could.

There was no cooking—that was out of the question—and we munched dry biscuits and washed them down with water from the cask—a liquid which possessed a rich brown colour, and tasted brackish. It was a novel experience, and my first night aboard that vessel was one that lives in my memory "ever-green.'

After three days of gale we got into the trades, which, though decidedly strong, are at least safe and steady, and by degrees I became acquainted with the "crew." The oldest of them had first of all run away to sea, but, following out the same lines, had deserted from his ship and that calling, and taken to singing and acting at an early age. His *confrére* was, if anything, of a more miserable type, and was an Irishman. These poor fellows had scarcely any clothing, and at last took to wearing their few tawdry stage costumes, "properties" which were easily carried about in an old battered wooden trunk. The effect of this was ludicrous enough. On the forecastle a shabby-genteel "stage gentleman" would be seen awaiting the signal to slip the jib-haulyards, his old worn tweed trousers, threadbare black tail-coat, and battered bowler hat contrasting very funnily with the surroundings. The other individual would be staggering along the weather deck, wildly endeavouring to grasp the main guy, dressed in a costume perfectly indescribable, but something between a Hamlet and a Mephistopheles. The skipper spent most of his time thrashing the boys, the woman in soothing the baby, which topped the gale with its shrieks and terrified all the albatrosses; whilst it gradually became understood that my presence was necessary at the wheel.

After twelve days of this kind of happy-go-lucky sailing, we found ourselves in shoals, and luckily, because whilst there was water enough for us to float over them, their presence enabled us to discover that we were 150 miles to the eastward of our course. This led to a hot debate on the depraved villainy which must have prompted the builders to send a vessel to sea so badly provided, on the uselessness of the shabby-genteel stage gentleman, and particularly of the Hamlet-cum-Mephistopheles hybrid, who always clutched the wrong ropes, and twice nearly brought the mainsail tumbling about our ears. It ended happily, however, the boys only getting a licking, and the skipper's emotion being thus calmed down.

It transpired by degrees why so curious an admixture of humanity had found its way aboard the "Toafa Haamia." They had exhausted the colony; no audience would go even to the lowest public-house parlour in Auckland to hear their miserable performance, and so the poor things had decided to emigrate to the Islands, and see if the guileless savage would appreciate their histrionic vagaries and part, in return, with his shining shilling. They agreed to work their passage, and thus the skipper secured the services of a free "crew."

How we ever reached Tonga, or why we got there, it would really be very difficult to honestly say. But, nevertheless, on the early morning of the fifteenth day land appeared on the port bow. It was still dark, though the stars shone brightly. No one was more astonished than the worthy skipper. It was evidently the first occasion upon which he had succeeded in striking his destination direct—the deviation of 150 miles being, from a sense of charity, left out of consideration. The land was low-lying, and a mist hung over it. The sea was smooth, but the swell dashed itself into foam when it came into contact with the coral reefs, as if angry at being suddenly stopped in its long, silent course. The breeze had fallen, and we drifted along, getting nearer and nearer to the shore. Then the sun rose, as it does in those tropical regions, with but a few minutes of dawn, and its powerful rays burst upon a scene of perfect novelty and beauty. Those rich, grassy flats, which run down to the coral beach, those stately cocoanut trees, those rustic huts nestling in the shade, and alive with a romantic dreamy race of beings, those perpetual blue skies and deep seas, with coral plants rearing their heads in the transparent water, presenting the appearance as the vessel drifts over them of a panorama—these are sights that cannot be painted in words or colours, cannot be reproduced in print or on canvas. It would be sacrilege to attempt a description of a land that is at once too beautiful to imagine, and too perfect to be thoroughly appreciated even by the visitor.

We drifted silently towards the harbour. A native pilot put off in a canoe and boarded us. He wore a straw hat with a green ribbon, a blue coat, a cloth tied round the waist, and nothing else. He took up his stand on the jib-boom, peering out for reefs, with the shabby-genteel stage gentleman close by, ready with his haulyard, his hat brushed, and a mouldy cravat carefully arranged. The Hamlet-cum-Mephistopheles curiosity clutched the main guy with the air of a tragedian who has at last thoroughly learned his part, and who is prepared to let go that rope when commanded, or die, like a Prince of Denmark, in the attempt. The skipper was aloft, straddling the crosstrees, and giving orders. The two lads were indulging in a pitched battle behind the foremast, their natural pugnacity finding full vent for the first time owing to the temporary absence of their stern commander. The woman was all smiles, the child had been washed and was crowing, and a thin, pale-faced individual stood at the wheel looking as if he had breathed nothing but London fog for the last ten years. All around were coral islands, and native canoes filled with men, women, boys, and girls coming off to gaze at the new arrivals.

There was the picture complete, and now is the time to ring down the curtain.

